# Supplementary material for: Comparative Transcriptome Analysis of Pine Trees Treated with Resistance-Inducing Substances against the Nematode Bursaphelenchus xylophilus
Source: Genes (Basel). 2020 Aug 26;11(9):1000. doi: 10.3390/genes11091000 (PMC7564552; doi:10.3390/genes11091000)
Supplement: Supplementary file 1 [file genes-11-01000-s001.zip › genes-886308-supplementary/Supplementary_figures.pdf]

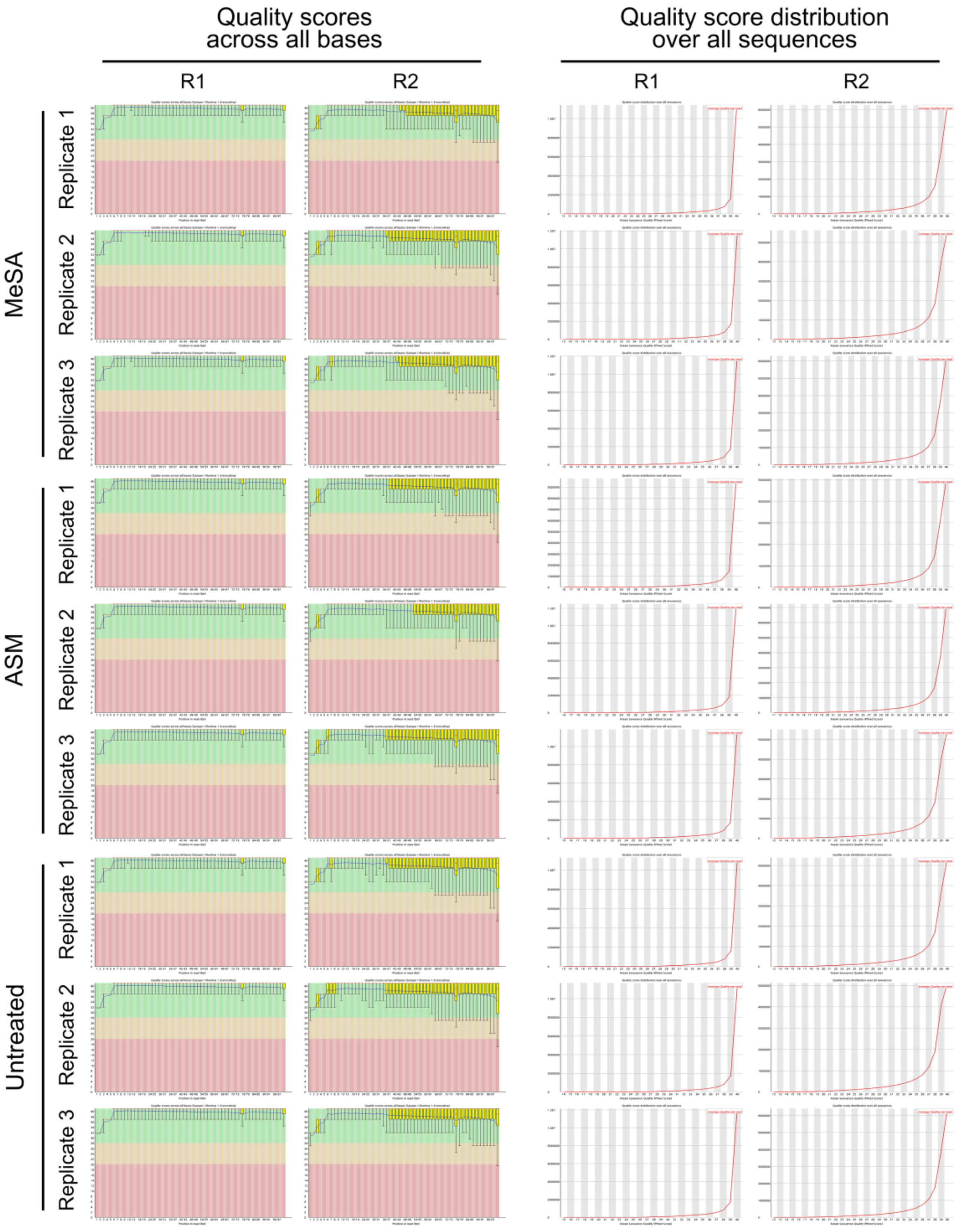

Figure S1. Quality assessment of raw reads in the FASTQ format. FastQC reports depicted the average quality scores of all reads for ASM, MeSA, and untreated samples. R1 is forward read and R2 is reverse read for pair-end sequencing. Box and whisker plots in the left panel display the distribution of per base quality for each of the 18 samples. The central red line indicates the median value, while the blue line represents the mean quality score. The yellow box displays the interquartile range (25%–75%), and the upper and lower whiskers represent the 10%–90% percentile. Graphical summary of average quality scores per read along the reads are shown in the right panel.

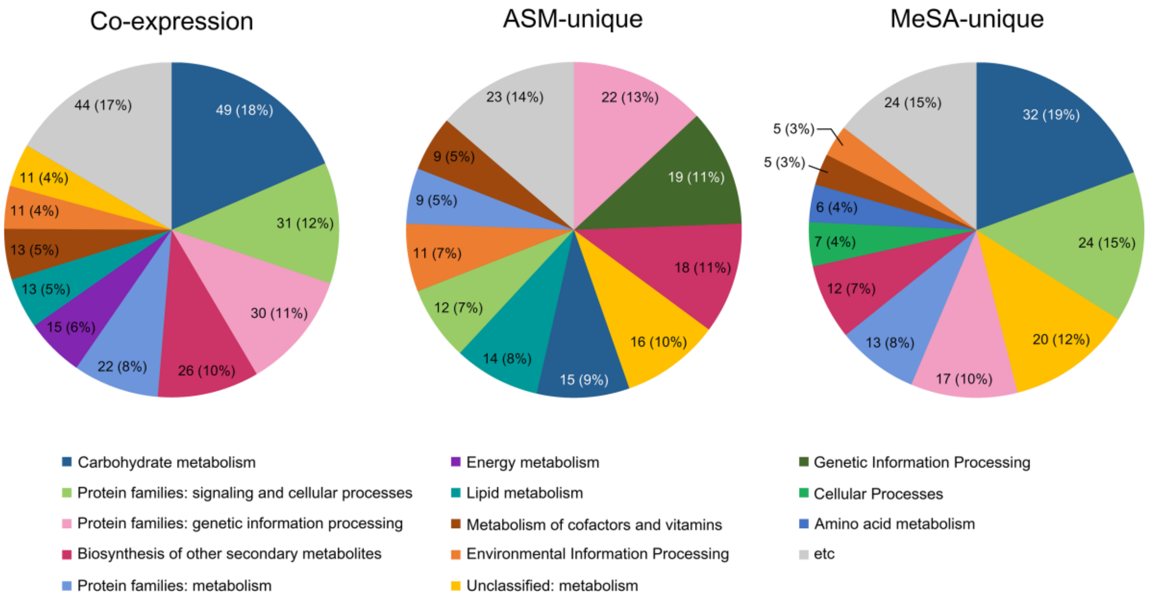

Figure S2. Pie charts showing functional categories according to BRITE hierarchy analysis. In the co-expression and unique patterns, functional annotations and their distribution are depicted as pie charts. The number of genes and relative proportions are indicated inside the chart.

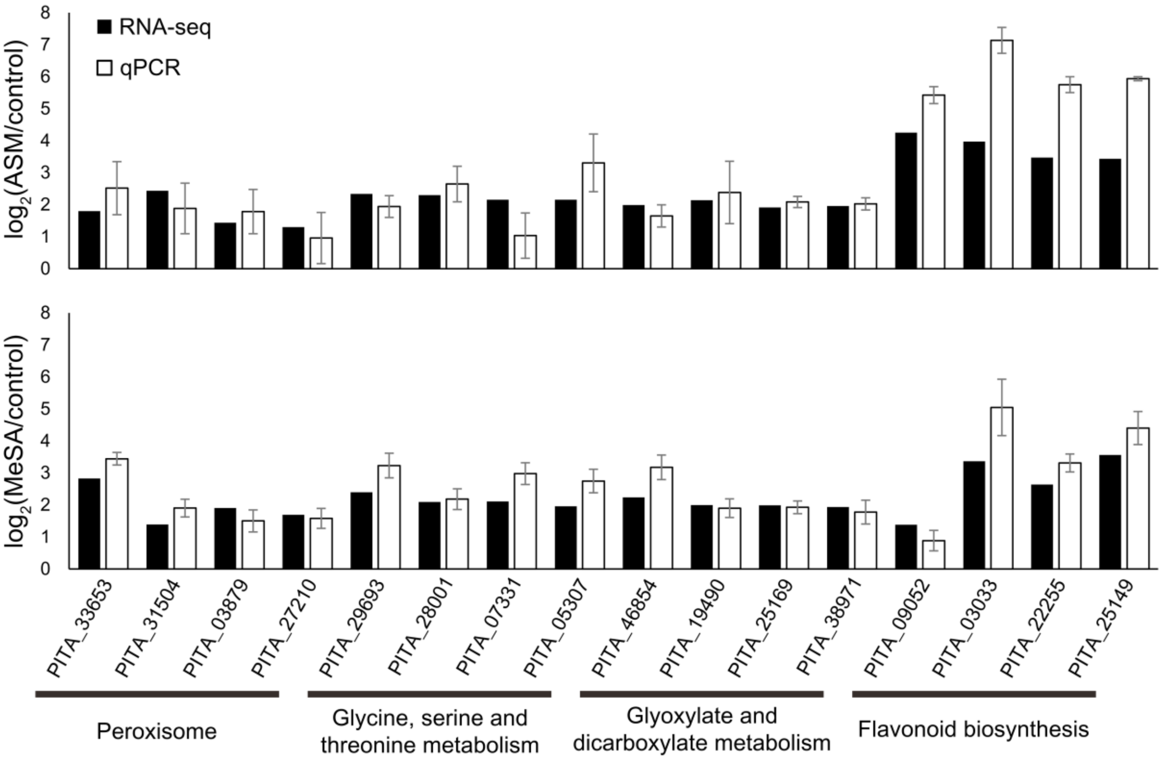

Figure S3. Verification of RNA-seq data by quantitative PCR (qPCR). The black bars represent fold changes based on RNA-seq data, according to the log_2(treatment/control) values. The gray bars with standard errors indicate fold changes based on qPCR data using the $2^{-\Delta\Delta CT}$ method for three biological replicates.

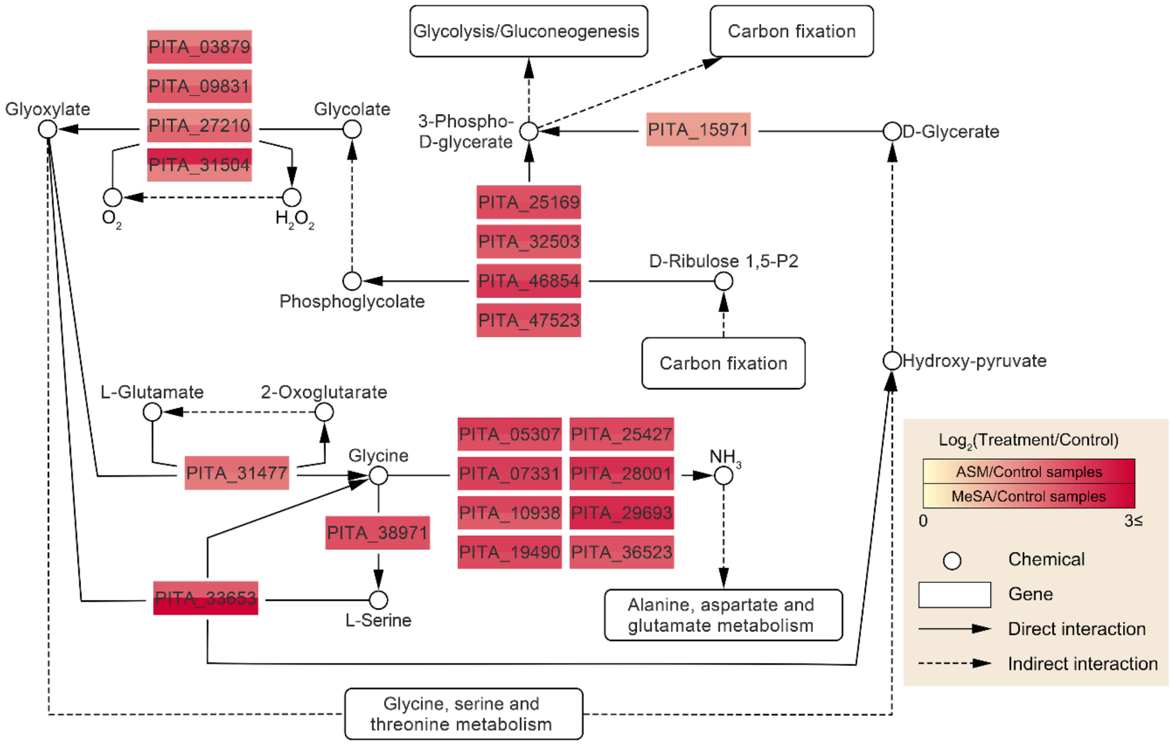

Figure S4. Molecular networks showing expression changes in the photorespiration module within glyoxylate and dicarboxylate metabolism. The nodes illustrate the type of molecules as follows: genes (coloured rectangles); biochemicals (circles); and cognate pathway (white rectangle). The differences in colour are indicative of the corresponding changes in log_2(treatment/control) values from a minimum of 0 to a maximum of 3. The figure is modified from KEGG map00630 using the Cytoscape tool.
